# Extended nursing and/or increased starter diet allowances for low weaning weight pigs

**DOI:** 10.5713/ajas.19.0511

**Published:** 2019-10-21

**Authors:** Aimee-Louise Craig, Ramon Muns, Alan Gordon, Elizabeth Magowan

**Affiliations:** 1 Sustainable Agri-Food Sciences Division, Agri-Food and Biosciences Institute, Hillsborough, Northern Ireland BT26 6DR, UK; 2 Sustainable Agri-Food Sciences Division, Agri-Food and Biosciences Institute, Newforge Lane, Belfast, Northern Ireland BT9 5PX, UK

**Keywords:** Starter Diet, Nurse Sow, Mortality, Light Weight Pigs, Weaning, Growth

## Abstract

**Objective:**

To evaluate the use of nurse sows and post-weaning nutrition strategies for low wean weight (WW) pigs on lifetime growth and efficiency.

**Methods:**

Animals (n = 270) were assigned to one of five treatments at 28 d. Low WW pigs (<6 kg) were either weaned and offered a special dietary regime recommended for low WW pigs (WEAN) or placed on a nurse sow (NURSE) and weaned at 49 d. Normal WW pigs (9 kg) (NORM) were also weaned at 28 d. After weaning, NORM and NURSE pigs were offered either a ‘high’ (4 kg/pig of starter 1 diet followed by 8 kg/pig of starter 2 diet) or ‘low’ (8 kg/pig of starter 2 diet) starter diet allowance in a 2×2 factorial arrangement. A typical grower diet was then offered, followed by a typical finisher diet until 147 d of age.

**Results:**

NORM pigs where heavier throughout their life compared to NURSE pigs (91.4 kg vs 76.2 kg at 147 d; p<0.001). WEAN pigs were heavier at 70 d compared to NURSE pigs (23.9 kg vs 21.0 kg; p<0.001), but there was no significant difference at 147 d between NURSE and WEAN treatments. NURSE pigs had reduced feed intake throughout the finishing period (1.6 kg/d; p<0.001) compared to WEAN (2.0 kg/d) and NORM (1.9 kg/d) pigs. Feed conversion ratio (FCR) of NURSE (2.20) was lower than NORM and WEAN during the finishing period (2.40 and 2.79, respectively).

**Conclusion:**

Extended (up to 49 d) nursing for low WW pigs resulted in improved FCR during the finishing period, but no overall improvement in growth rate compared to low WW pigs weaned at 28 d and offered a specialised starter regime. Normal WW pigs where significantly heavier than low WW pigs throughout the study.

## INTRODUCTION

In modern pig production, low weaning weight pigs present a chronic problem due to the use of hyperprolific sows [1]. Low weight pigs at weaning have immature digestive systems, are slower growers, tend to have higher mortality, and poorer carcass value than their heavier counterparts [2,3]. There is limited literature on rearing strategies for low weight pigs both pre- and post-weaning [4,5]. Extended suckling has the potential to improve gut maturity at weaning [6], but research on using nurse sows has mainly focused on sow and piglet welfare [7,8] rather than on piglet performance. Increased amounts of starter diets have been found to improve the performance of low weaning weight pigs [9–11]. The present study is the first, known to the authors, investigating the effect of different management systems on life-time performance of low WW pigs (<6 kg at 28 d). Therefore, the objective of the study was to evaluate the implementation of nurse sows and different starter diet allowances post-weaning and their impact on the growth of disadvantaged pigs.

## MATERIALS AND METHODS

The trial was conducted at Agri-Food and Biosciences Institute (AFBI), Hillsborough, Northern Ireland. All experimental procedures were conducted under an experimental licence (no. 2751) granted by the Department of Health, Social Services and Public Safety for Northern Ireland in accordance with the Animals (Scientific Procedures) Act 1986. A total of 270 piglets (PIC 337×[Large White×Landrace]) from 24 sows were selected at 28 d of age (end of lactation).

### Animals and housing

At d 109 of gestation, sows were moved to a climate controlled (21.0°C) farrowing room and allocated to individual farrowing crates (0.5×2.2 m), each one placed at the centre of a farrowing pen (2.3×1.5 m). Sows had access to a wet and dry feeder and were fed a commercial lactation diet (13.5 MJ digestible energy (DE)/kg, 94.4% dry matter, 17% crude protein (CP), 3.6% crude fibre (CF), and 1% total Lys). On the farrowing day, sows were offered 2.5 kg of the lactation diet, and the amount offered increased by 0.5 kg daily until 10 kg/d was reached. The target average feed intake over 28 d lactation was 8 kg/d. Attached to the front of each pen, there was a shelter box (1.5×0.6 m) for piglets equipped with a heated floor pad (heated forward creep area). Temperature in the farrowing rooms and creep areas was electronically controlled. Within 12 hours of birth all piglets had their teeth clipped, tails docked and received 2 mL of an iron supplement (Uniferon; Virbac Ltd., Suffolk, UK). Piglets were also tagged to allow recording of individual animals. No creep feed was offered to the piglets, and sow troughs were 45 cm high preventing piglets from easy access to sow feed. At d 28 piglets were vaccinated for M. Hyo and PCV2 with Ingelvac MycoFLEX and Ingelvac CircoFLEX (Boehringer Ingelheim Ld., Bracknell, UK), respectively. Cross-fostering of piglets between litters was performed within the first 24 h after birth to ensure even litter sizes (av. 12.5 piglets/sow). Piglets were weaned at 28±2 d of age.

At weaning, pigs were transferred to nursery accommodation (stage 1 and stage 2 combined) with plastic slatted floors (0.38 m^2^/pig). Temperature was 28°C on day one and was reduced by 0.5°C/d to a minimum of 18°C. Pigs were offered feed via a small circular hopper (Rotecna S.A, Agramunt, Spain) for the first week post weaning. Thereafter, pigs were offered feed in a ‘dry multi-space feeder’ (Etra Feeders Ltd., Dungannon, UK) with a feeder space allowance of 6.6 cm/pig. At 70 d of age, pigs were transferred to finishing accommodation with fully slatted concrete floors (0.61 m^2^/pig). In the finishing accommodation pigs were offered feed and water via a ‘wet and dry’ single space shelf feeder (Etra Feeders Ltd., UK) and 1 bowl drinker. One feeder was placed per pen (10 pigs/pen).

### Treatments and dietary regime

A total of 180 low weight pigs (5.12±0.780 kg) and 90 normal weight pigs (NORM, 8.92±0.415 kg) were used in the experiment. The trial was carried out over nine time periods or production batches. In each batch, 30 piglets were selected at weaning and allocated to one of the five treatments. Low weight and NORM pigs were distributed to treatments balancing for gender and parity of origin. Low weight pigs were either weaned (WEAN, n = 90) and offered a special dietary regime recommended for low wean weight pigs (1 kg/pig of special starter diet [18.0 MJ DE/kg, 20.8% CP, 13.5% oils and fats, 2.0% CF, and 1.8% total Lys; Devenish Nutrition Ltd., Belfast, UK], 4 kg/pig of starter 1 diet [6.5 MJ DE/kg, 22.5% CP, 8.5% oils and fats, 2.5% CF, and 1.7% total Lys; Devenish Nutrition Ltd., UK] and 8 kg/pig of Starter 2 diet [16.0 MJ DE/kg, 22.0% CP, 8.3% oils and fats, 3.5% CF, and 1.6% total Lys; Devenish Nutrition Ltd., UK]) or placed on a nurse sow (NURSE, n = 90) and weaned at 49 d of age (weaned with the following production batch). No creep feed was offered during the extended suckling period to avoid further confounding of treatment. At weaning, NURSE pigs were offered either ‘high’ (4 kg/pig of starter 1 diet followed by 8 kg/pig of starter 2 diet; NURSE ‘high’, n = 45) or ‘low’ (8 kg/pig of starter 2 diet; NURSE ‘low’, n = 45) starter diet allowances. NORM pigs were weaned at 28 d of age and offered either ‘high’ (n = 45) or ‘low’ (n = 45) starter diet allowances as described above. Sows with a body condition score of at least 2.5 (on a 5-point scale where: 1 = very thin to 5 = overfat) and an average feed intake of 7.5 kg/d over a 28 d lactation, and with a litter size of at least 10 piglets at weaning were selected as nurse sows. Any sow that nursed experimental pigs during the conventional lactation was discarded as a nurse sow.

After pigs had consumed their respective starter allowances (average time: WEAN 4 wk, NORM ‘high’ 3.5 wk, NORM ‘low’ and NURSE ‘high’ 3 wk, NURSE ‘low’ 2.5 wk), they were offered *ad libitum* access to grower diets (13.9 MJ DE/kg, 18.8% CP, 4.2% oils and fats, 2.6% CF, and 1.2% total Lys) until they reached 84 d of age. Pigs were then offered a finishing diet (13.4 MJ DE/kg, 17.1% CP, 3.3% oils and fats, 3.6% CF, and 1.0% total Lys) until the end of the trial (147 d of age). On transfer to the finishing house, NURSE ‘low’ and ‘high’ groups were combined into one pen of 10 pigs and the NORM ‘low’ and ‘high’ groups were also combined into one pen of 10 pigs due to housing limitations. Due to the extended lactation, NURSE pigs (both ‘high’ and ‘low’) were 21 d older than their counterparts when transferred to the finishing accommodation and offered the finishing diet (105 d of age instead of 84 d).

### Measurements

Pigs were individually weighed and pen feed intakes recorded at weaning (28 d), 49, 70, 84, 105, 126, and 147 d of age (irrespective of the treatment). Average daily gain (ADG) was calculated on an individual basis, while average daily feed intake (ADFI) was calculated on a pen basis as: feed consumed divided by the number of pigs per pen. Food conversion ratio (FCR) was also calculated on a pen basis as: food consumed per pen divided by the weight gained within the pen. Mortality was calculated as the percentage of pigs that died or were euthanized in each pen. Blood samples were taken from the jugular vein of a subset of pigs (n = 10/treatment over two time periods) at 1 day pre-weaning (NURSE pigs were 48 d old, and WEAN and NORM were 27 d old, respectively), and again at 14 and 28 d post-weaning (NURSE pigs were 63 d and 77 d old and WEAN and NORM pigs were 42 d and 56 d old, respectively). Serum was extracted by centrifugation (1509.3G force for 15 minutes at 18°C) and frozen (−20°C) until analysis was performed. Analysis was carried out using a commercially available ELIZA Quantitation Set (Cat. No. E100-104, Bethly Laboratories Inc., Montgomery, TX, USA) to test for immunoglobulin G (Ig G) concentration in porcine serum.

### Statistical analysis

Individual pig performance data (body weight, ADG, feed intake, FCR, and mortality) was analysed using the linear mixed model methodology (with pen as the statistical unit) with time period fitted as a random effect using the model: Y = μ+sow+diet+sow×diet+e (sow = NORM, NURSE; diet = high, low). A post-hoc t-test was used to assess the effect of NURSE vs WEAN. Production batch was introduced as fixed effect. Data recorded during the finishing period (feed intake and FCR) was analysed on a pen basis using a one-way analysis of variance with three treatments: WEAN, NURSE, and NORM. No covariates were applied. Serum IgG concentrations were analysed using a general linear model with Treatment introduced as a main effect, and production batch introduced as a fixed effect. IgG concentration at 1 d pre-weaning was introduced as a co-variate in the models for IgG concentration at 14 and 28 d post-weaning. All analyses were carried out using Genstat 16th Edition (Lawes Agriculutral Trust, Rothamsted Experimental Station). Pig growth for each treatment was plotted with a simple linear regression with groups using the model y(wt) = ax+b to predict days to a standard weight of 100 kg.

## RESULTS

### Performance from 28 to 70 days of age

No interactions between NURSE/NORM and high/low dietary regimes were tested between 28 and 49 d of age because NURSE pigs were still suckling. However, the analysis comparing NORM ‘high’ and NORM ‘low’ showed that at 49 d of age, NORM ‘high’ was heavier (p<0.001) than NORM ‘low’ (17.6±2.29 vs 14.7±2.38 kg). In addition, at 49 d of age the body weight of WEAN (12.3±2.47 kg) was higher than NURSE (10.6±1.68 kg) and lower than NORM (16.2±2.38 kg) (p< 0.001, respectively); while body weight of NURSE was lower than NORM (p<0.001).

The live weight of WEAN pigs was greater at 28 d and 70 d of age than the NURSE pigs (Table 1; p<0.001). ADG from 49 to 70 d of age was also greater for WEAN pigs than NURSE pigs (p<0.05). NORM pigs were on average 8 kg heavier than NURSE pigs (p<0.001) at 70 d of age. Pigs on the ‘high’ starter diet allowance were heavier (p<0.001) and had greater ADG from 49 to 70 d of age (p<0.001) than those on the ‘low’ starter diet allowance.

From 49 to 70 d of age WEAN pigs ate more than NURSE pigs (Table 2; p<0.001). Pigs on the ‘high’ starter allocation ate more than those on the ‘low’ allocation from 49 to 70 d of age (p<0.05). NORM pigs also ate more than the NURSE pigs (p<0.001) between 49 to 70 d of age. Pen FCR from 49 to 70 d of age (Table 2) was improved when NURSE pigs were offered the ‘low’ starter diet allowance, but not when NORM pigs were offered the ‘low’ starter diet allowance (p<0.05). WEAN pigs had increased FCR from 49 to 70 d of age compared to NURSE pigs (1.80 vs 1.16; p<0.001). From 49 to 70 d of age, pigs offered the ‘high’ starter allowance had increased FCR compared to pigs offered ‘low’ starter allowance (1.42 vs 1.40; p<0.05). Also from 49 to 70 d of age, NURSE pigs had lower FCR than NORM pigs (1.16 vs 1.67; p<0.001).

### Performance from 70 to 147 days of age

There were no interactive effects between treatments on live weight and ADG in the finishing stage (Table 1). Live weights and ADG for NORM pigs were consistently higher than NURSE and WEAN pigs. WEAN pigs were heavier than NURSE pigs at 84 d of age (p<0.001), but not at 105, 126, or 147 d of age. ADG of WEAN and NURSE pigs did not differ between 84 to 147 d of age (p>0.05). Pigs that had ‘high’ starter diet allocations were consistently heavier throughout the finishing period (p<0.001) and had greater ADG from 84 to 147 d of age (p<0.05) than those offered the ‘low’ allocation of starter diets.

No differences were found in ADFI between treatments from 70 to 126 d of age (p>0.05, Table 3). From 126 to 147 d of age, NURSE pigs consumed less than the other treatments (p<0.001). NURSE pigs had a lower overall intake from 70 to 147 d of age than NORM and WEAN treatments (p<0.001). Throughout the finishing period, NURSE pigs had a consistently lower FCR than WEAN pigs (p<0.001) except from 70 to 84 d of age (p>0.05). From 70 to 147 d of age NURSE pigs had lower FCR than both WEAN and NORM pigs (p< 0.001).

### Prediction of growth to 100 kg

The plotted linear regression indicated that the growth curves for each treatment were distinct (p<0.001; R^2^ = 0.89). The equations for each treatment are as follows (liveweight, LWT; SE, standard error):

NORM high:
  LWT=-7.872 (SE 1.050)+0.6408 (SE 0.010622)×day
NORM low:
  LWT=-7.597 (SE 1.029)+0.5796 (SE 0.010188)×day
NURSE high:
  LWT=-9.131 (SE 1.042)+0.5522 (SE 0.010514)×day
NURSE low:
  LWT=-8.547 (SE 1.084)+0.5115 (SE 0.011129)×day
WEAN:
  LWT=-8.242 (SE 0.811)+0.5381 (SE 0.007399)×day

**Figure 1:**
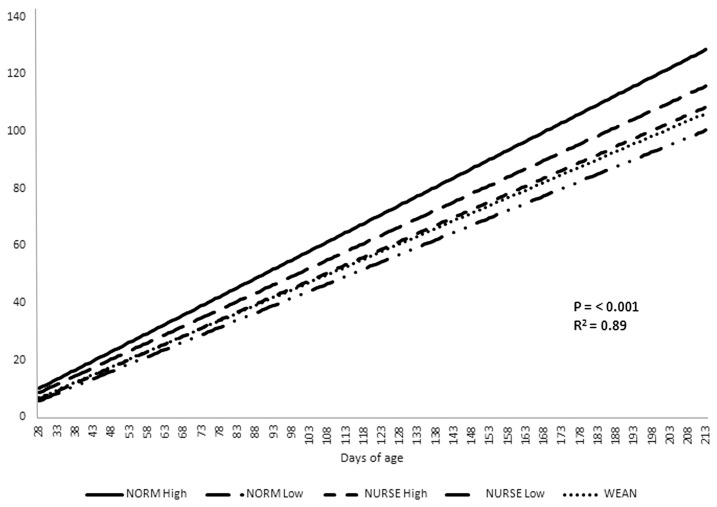
Predicted days to 100 kg liveweight. NORM high, normal weight pigs (8.9±0.41 kg) weaned at 28 d of age, then offered 4 kg/pig of starter 1 diet followed by 8 kg/pig of starter 2 diet at weaning; NORM low, normal weight pigs (8.9±0.41 kg) weaned at 28 d of age, then offered 8 kg/pig of Starter 2 diet at weaning; NURSE high, low weight pigs (5.1±0.78 kg) placed on a nurse sow and weaned at 49 d of age, then offered 4 kg/pig of Starter 1 diet followed by 8 kg/pig of starter 2 diet at weaning; NURSE low, low weight pigs (5.1±0.78 kg) placed on a nurse sow and weaned at 49 d of age, then offered 8 kg/pig of starter 2 diet at weaning; WEAN, low weight pigs (5.1±0.78 kg) weaned at 28 d of age and offered 1 kg/pig of special starter diet, 4 kg/pig of starter 1 diet, and 8 kg/pig of starter 2 diet.

Using these equations, time to 100 kg was 169 d, 186 d, 198 d, 212 d, and 201 d for NORM high, NORM low, NURSE high, NURSE low, and WEAN, respectively (Figure 1).

### Mortality and serum IgG concentrations

Pig mortality between 28 to 70 d of age was higher in NURSE pigs than WEAN pigs (Table 2; p = 0.001) and was 0% for NORM pigs. There was no significant effect of starter diet allowance on pig mortality during from 28 to 70 d of age. From 70 to 147 d of age there was no significant difference between treatments. There was an interaction between the lifetime mortality of NURSE and NORM pigs on the ‘high’ and ‘low’ starter diets (p<0.05). NORM pigs had higher mortality on the ‘high’ starter diet allocation than on the ‘low’, whereas NURSE pigs had lower mortality on the ‘high’ starter diet allocation than on the ‘low’ (p<0.05, respectively).

**Figure 2:**
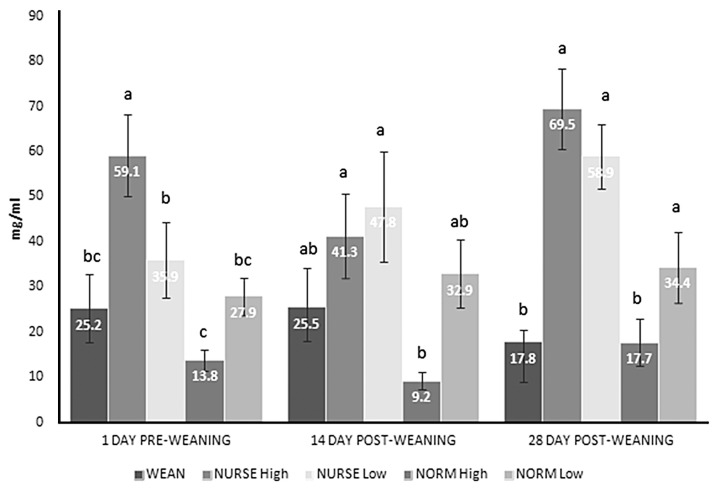
Serum immunoglobulin G (Ig G) concentrations across treatments. WEAN, low weight pigs (5.1±0.78 kg) weaned at 28 d of age and offered 1 kg/pig of special starter diet, 4 kg/pig of starter 1 diet, and 8 kg/pig of starter 2 diet; NORM high, normal weight pigs (8.9±0.41 kg) weaned at 28 d of age, then offered 4 kg/pig of starter 1 diet followed by 8 kg/pig of starter 2 diet at weaning; NORM low, normal weight pigs (8.9±0.41 kg) weaned at 28 d of age, then offered 8 kg/pig of Starter 2 diet at weaning; NURSE high, low weight pigs (5.1±0.78 kg) placed on a nurse sow and weaned at 49 d of age, then offered 4 kg/pig of starter 1 diet followed by 8 kg/pig of starter 2 diet at weaning; NURSE low, low weight pigs (5.1±0.78 kg) placed on a nurse sow and weaned at 49 d of age, then offered 8 kg/pig of starter 2 diet at weaning. ^a–c^ Bars with different superscripts differ (p<0.05).

Figure 2 shows the serum IgG concentration for each treatment group. At 1 d pre-weaning NURSE ‘high’ had higher IgG concentration than the other treatment groups (p<0.05, respectively), NURSE ‘low’ was higher than NORM ‘high’ (p<0.05). At 14 d post-weaning, NURSE ‘low’ and ‘high’ had both higher IgG concentration than NORM ‘high’ (p<0.05, respectively). At 28 d post-weaning WEAN and NORM ‘high’ had both lower IgG concentration than NORM ‘low’ (p<0.05, respectively), and lower than NURSE ‘high’ and NURSE ‘low’ (p<0.001, respectively).

## DISCUSSION

On commercial farms pigs are being weaned as light as 3 kg at 28 d and a wean weight of 7.5 kg is a common average wean weight at 28 d of age [12]. To better investigate the performance of disadvantaged pigs (5.12±0.780 kg of body weight) through to the end of the finishing period (147 d of age), NORM pigs (8.9±0.41 kg) were included in the study to set a standard against which any improvements could be compared.

At weaning, the resultant weight of pigs on the WEAN treatment was higher than those on the NURSE ‘low’ treatment. Initially, low weight pig treatments (WEAN, NURSE ‘high’ and ‘low’) were analysed separately from the NORM treatments and weaning weight was introduced as covariate in the models to correct for the initial difference. The growth pattern and treatment differences observed were the same as with the inclusion of NORM pigs in the analysis. Therefore, although the initial difference in weaning weight has been considered in the discussion, it was not considered to invalidate the interpretation of the statistic outcome. It is also recognised that combining both NURSE treatments into one pen, and both NORM treatments into one pen at the start of the finishing period, may have potential for bias in terms of feed intake and FCR. Therefore, the effect of ‘high’ and ‘low’ starter diet on the finishing period have not been discussed. Nonetheless, the results are considered relevant to the scientific and commercial audience and the work has been completed to ensure treatments were properly compared.

### Impact on growth performance

At 70 d of age, pigs on the WEAN treatment had a 12.7% weight advantage compared to NURSE pigs (23.9 vs 21.0 kg). However, NURSE pigs were still suckling during the first half of this period, with no access to creep feed, while WEAN pigs were being offered solid diets (more nutrient dense than milk). Therefore, it would be recommended to offer creep feed to piglets suckling nurse sows to enhance their feed intake post-weaning and subsequent growth [9,11].

In the WEAN treatment, pig ADG was reduced by 164 g/d between 84 to 105 d of age which coincided with the change from grower to finisher diet. This suggests that these pigs, weighing 34 kg on average, were not ready for the finisher diet. Previous research [13] also found that a similar finisher diet (18.6 MJ gross energy, 0.8 g/kg available lysine) was inadequate for pigs less than 40 kg. However, in treatments NORM (45 kg) and NURSE (40 kg) ADG was also reduced by 72 g/d and 102 g/d in the 21 d period after the finisher diet was introduced. This suggests that even pigs of 40 kg where not ready for the finisher diet. It has been suggested previously that it is economically viable to offer a grower diet up to 60 kg of live weight to optimise performance [14]. Indeed evidence from this trial suggests that introducing the finisher diet based on live weight (perhaps up to 60 kg) would be more beneficial than based on age.

As expected, the live weight of the NORM pigs was greater than the low weight pig treatments throughout the growing and finishing periods. Weaning weight plays a critical role in growth [15] and has been suggested as a better predictor of future growth than birth weight [16].

### Feed conversion ratio and feed intake

The NURSE piglets had improved FCR compared to both the NORM and WEAN pigs throughout their post-weaning life. This may be explained by the fact NURSE pigs had lower intake (average of 1.6 kg/d) from 70 to 147 d of age compared to WEAN and NORM groups (overall av. of 1.9 kg/d). NURSE pigs were offered higher specification diets for their age due to older weaning age. It is known that diets with higher nutrient densities improve FCR [17]. When NORM and NURSE treatments were both offered the finisher diet at the same time (from 105 to 147 d of age), FCR did not differ statistically. It is also possible that the extended suckling period may have improved gut function [6] and contributed to the reduced post-weaning growth check.

In other studies, offering a high allowance of starter diet improved pig growth, especially for low wean weight pigs [10, 11,18]. However, in contrast to these studies there was no increase in feed intake to compensate for low nutrient specification in the low allowance in this study. It could be because the difference between high and low allocations were more severe and therefore less palatable for piglets offered the Starter 2 diet immediately post-weaning. The rationale behind this design was that the older piglets coming off a nurse sow at 49 d may have been able to tolerate the lower specification Starter 2 diet. Indeed, the difference in performance between the high and low treatments in NURSE pigs was less, although not significant, than the high and low treatments for NORM pigs both at 70 d (2.4 kg vs 4.4 kg) and 147 d weeks (4.6 kg vs 9.2 kg); therefore if NORM weight pigs had been NURSE treated their growth and efficiency could be hypothesised to be greater. It has been noted historically that suckling pigs of normal weight, when offered an *ad libitum* milk diet can reach ADG of over 0.5 kg/d [19]. With modern genetics, NORM pigs should have even greater growth potential than has been realised, but their growth quickly outstrips the sow’s milk yield.

### Mortality and serum IgG concentrations

NURSE treatments had greater mortality in the nursery period (up to 70 d of age) than in the finishing period. However, for WEAN and NORM ‘high’ treatments there was very little mortality in the nursery, while over 7.5% of pigs died in the finishing house. Poor thrift was the main cause of death, although all pigs were vaccinated for PCV 2. The pattern of mortality had an impact on economics. Economically, the NORM treatments had the highest margin over feed cost. This is expected as 8.9 kg pigs at 28 d are likely to achieve better 147 d weights and be more efficient than small pigs [15]. Despite the added cost of feeding a sow for 21 d, feed costs for NURSE treatment was lower than for WEAN pigs (per 10 pigs). WEAN pigs had higher mortality later in the finishing house, which likely contributed to their lower economic value as the expensive starter diets had been eaten by pigs who did not reach slaughter. Calculating the age at which the treatments would reach a target of 100 kg live weight from their individual treatment growth curves, NURSE High would have finished 3 d before WEAN pigs, but 14 d quicker than NURSE low. In comparison, NORM high and low pigs would have finished 29 and 12 d before NURSE high. Therefore, from an economic perspective and also from a pig throughput perspective, light weaning weight pigs allowed to suckle a sow for a further 21 d, and then offered a High allocation of starter diets perform better than pigs weaned and offered a three starter diet regime.

As expected, NURSE pigs (both ‘high’ and ‘low’) had higher concentrations of serum IgG than the other groups in the 3 measuring points, indicating improved humoral immunity [20], mainly due to being of older age at each measuring point. Surprisingly, NORM ‘high’ pigs had significantly lower IgG concentration than NORM ‘low’ at 28 d post-weaning, and did not differ from WEAN pigs at any sampling point. In contrast, a different study [21] found lower IgG concentration in low (≈ 5 kg) wean weight pigs compared to normal (≈ 7 kg) wean weight pigs. Our findings might suggest that, at the same age, small pigs might benefit from a higher quality diet post-weaning, whereas in normal weight pigs a ‘high’ starter diet allowance does not enhance their immunological maturity compared to a ‘low’ allowance. More research is needed to understand the impact of starter diet and its allowance on piglets’ active immune capacity based on their weaning weight.

## CONCLUSION

A weight difference of 3.8 kg at weaning increased to 7.4 kg and 13.8 kg at 70 and 147 d respectively between the NORM (8.9±0.41 kg) and light weight pigs (5.1±0.78 kg). Low weight pigs allowed to suckle a nurse sow until 49 d of age and then offered ‘high’ allowance of starter diet and a later transition to the finisher diets (105 d of age), had similar weight at 147 d of age but an improved finisher FCR (2.20 vs 2.79) compared to low weight pigs weaned at 28 d and offered a high specification dietary regime. The present study has identified the opportunity to improve the lifetime FCR of low weaning weight pigs by extending the lactation period using nurse sows. Nonetheless, further research is needed to better understand the implications for mortality and to identify the most efficient post-weaning feeding strategy.

## Figures and Tables

**Table 1:** Effect of treatment on piglet lifetime live weight and weight gain

Items	WEAN1)	NURSE2)		NORM3)		SED	p-values			
		
		High4)	Low5)	High	Low		WEAN vs NURSE	High vs low	NURSE vs NORM	Interactions6)
n° of pigs/n° of pens7)	90/9	45/9	45/9	45/9	45/9					
Live weight (kg)										
28 d of age	5.5	5.1	4.8	8.9	8.9	0.1	<0.001	0.198	<0.001	0.147
49 d of age8)	12.3	10.8	10.4	17.6	14.7	-	-	-	-	-
70 d of age	23.9	22.2	19.8	31.6	27.3	0.8	<0.001	<0.001	<0.001	0.116
84 d of age	33.8	32.3	29.2	42.9	36.9	1.2	<0.001	<0.001	<0.001	0.277
105 d of age	45.2	49.1	44.6	56.7	50.1	1.7	0.208	<0.001	<0.001	0.404
126 d of age	59.3	63.2	58.4	72.3	65.1	2.3	0.369	<0.001	<0.001	0.484
147 d of age	79.9	78.5	73.9	96.0	86.8	2.8	0.084	<0.001	<0.001	0.274
Average daily gain (g/d)										
49 to 70 d of age	551	542	450	669	597	28	0.009	<0.001	<0.001	0.634
70 to 84 d of age	712	723	668	772	686	39	0.560	0.015	0.289	0.595
84 to 105 d of age	548	798	732	684	630	40	<0.001	0.045	<0.001	0.842
105 to 126 d of age	693	676	651	757	716	48	0.409	0.357	0.050	0.836
126 to 147 d of age	990	721	740	1,102	1,029	54	<0.001	0.459	<0.001	0.251
84 to 147 d of age	743	734	701	834	773	30	0.280	0.034	<0.001	0.539

SED, standard error of difference.

1) WEAN = low weight pigs (5.1±0.78 kg) weaned at 28 d of age and offered 1 kg/pig of special starter diet, 4 kg/pig of starter 1 diet, and 8 kg/pig of starter 2 diet.

2) NURSE, low weight pigs (5.1±0.78 kg) placed on a nurse sow and weaned at 49 d of age.

3) NORM, normal weight pigs (8.9±0.41 kg) weaned at 28 d of age.

4) High, pigs offered 4 kg/pig of starter 1 diet followed by 8 kg/pig of starter 2 diet at weaning.

5) Low, pigs offered 8 kg/pig of starter 2 diet at weaning.

6) Interaction between starter diet allowance (high/low) and type of pig (NURSE/NORM).

7) From d 70 to 147, NURSE and NORM groups were combined into 10 pig pens.

8) No statistical analysis as NURSE pigs had not been offered high or low starter treatments.

**Table 2:** Effect of treatment on feed intake and feed conversion ratio of nursery pigs

Items	WEAN1)	NURSE2)		NORM3)		SED	p-values			
		
		High4)	Low5)	High	Low		WEAN vs NURSE	High vs low	NURSE vs NORM	Interactions6)
n° of pigs/n° of pens7)	90/9	45/9	45/9	45/9	45/9					
ADFI (g/d)										
49 to 70 d of age	987	678	599	1,138	985	47.9	<0.001	0.002	<0.001	0.281
FCR										
49 to 70 d of age	1.80	1.17	1.14	1.67	1.66	0.057	<0.001	0.009	<0.001	0.006
Mortality (%)										
28 to 70 d of age	1.3	4.2	7.1	0.0	0.0	1.38	<0.001	0.158	<0.001	0.148
70 to 147 d of age	10.7	2.2	6.7	7.8	0.0	4.53	0.117	0.606	0.863	0.064
28 to 147 d of age	12.1	6.7	15.6	7.8	0.0	5.17	0.935	0.763	0.040	0.040

SED, standard error of difference; ADFI, average daily feed intake; FCR, food conversion ratio.

1) WEAN, low weight pigs (5.1±0.78 kg) weaned at 28 d of age and offered 1 kg/pig of special starter diet, 4 kg/pig of starter 1 diet, and 8 kg/pig of starter 2 diet.

2) NURSE, low weight pigs (5.1±0.78 kg) placed on a nurse sow and weaned at 49 d of age.

3) NORM, normal weight pigs (8.9±0.41 kg) weaned at 28 d of age.

4) High, pigs offered 4 kg/pig of starter 1 diet followed by 8 kg/pig of starter 2 diet at weaning.

5) Low, pigs offered 8 kg/pig of starter 2 diet at weaning.

6) Interaction between starter diet allowance (high/low) and type of pig (NURSE/NORM).

7) From d 70 to 147, NURSE and NORM groups were combined into 10 pig pens.

**Table 3:** Effect of treatment on finishing pig performance

Items	WEAN1)	NURSE2)	NORM3)	SED	p-value
n° of pigs/n° of pens	90/9	85/9	90/9		
ADFI (g/d)					
70 to 84 d of age	1,394	1,210	1,322	54.4	0.086
84 to 105 d of age	1,528	1,459	1,567	55.5	0.404
105 to 126 d of age	1,936	1,552	1,874	109.0	0.052
126 to 147 d of age	2,948^b^	1,978^a^	2,694^b^	102.2	<0.001
70 to 147 d of age	1,988^b^	1,579^a^	1,904^b^	44.6	<0.001
FCR					
70 to 84 d of age	2.11	1.73	1.90	0.151	0.241
84 to 105 d of age	2.87^c^	1.91^a^	2.49^b^	0.125	<0.001
105 to 126 d of age	2.95^b^	2.37^a^	2.63^a^	0.100	0.003
126 to 147 d of age	3.08^b^	2.70^a^	2.53^a^	0.077	<0.001
70 to 147 d of age	2.79^c^	2.20^a^	2.40^b^	0.062	<0.001

SED, standard error of difference; ADFI, average daily feed intake; FCR, food conversion ratio.

1) WEAN, low weight pigs (5.1±0.78 kg) weaned at 28 d of age.

2) NURSE, low weight pigs (5.1±0.78 kg) placed on a nurse sow and weaned at 49 d of age.

3) NORM, normal weight pigs (8.9±0.41 kg) weaned at 28 d of age.

a–c Means within a row with different superscripts differ (p<0.05).
